# Physiological and locomotor variations of 3v3 and 5v5 small-sided games soccer formats: A 4-month study on sedentary young adults

**DOI:** 10.1177/00368504231224606

**Published:** 2024-01-09

**Authors:** Qi Xu, Rui Miguel Silva, Kai Qi, Dong Ma, TingYu Li, Filipe Manuel Clemente

**Affiliations:** 1 Department of Biomechanics and Sport Engineering, Gdansk University of Physical Education and Sport, Gdańsk, Poland; 2 Research Center in Sports Performance, Recreation, Innovation and Technology (SPRINT), Melgaço, Portugal; 3 Escola Superior Desporto e Lazer, 112031 Instituto Politécnico de Viana do Castelo, Rua Escola Industrial e Comercial de Nun’Álvares, Viana do Castelo, Portugal; 4 Instituto de Telecomunicações, Delegação da Covilhã, Lisboa, Portugal

**Keywords:** football, recreational populations, small-sided games, variability

## Abstract

The objectives of this study were twofold: (a) to analyze the variability of 3v3 and 5v5 small-sided games (SSG) formats in sedentary young adults, and (b) to compare the physiological and locomotor demands of 3v3 and 5v5 SSG formats while considering variations based on sex. The study followed a longitudinal design over 4 months. Thirty sedentary young adults with a mean age of 20.2 ± 1.0 years, height of 1.67 ± 0.06 m, and body mass of 86.3 ± 11.8 kg were included in the study. The participants engaged in 3v3 and 5v5 SSG formats, with each format being played 10 times per month. During each session, heart rate (HR), rate of perceived exertion (RPE), and total distance were measured and analyzed. The within-player variability for HR ranged from a minimum of 1.6% to a maximum of 2.8% (considering the levels at each month), while the between-players variability for HR ranged from a minimum of 1.4% to a maximum of 2.6% (considering the levels at each month). Similar variability patterns were observed for the other outcomes. In terms of comparisons between the formats, the 3v3 format resulted in higher RPE than the 5v5 format for both male (*p* = 0.006) and female (*p* = 0.628) participants, as well as for the other outcome measures. In summary, these findings highlight the reproducibility of physiological responses in 3v3 and 5v5 SSG among sedentary individuals. Notably, the 3v3 format consistently induced higher RPE levels. These findings underscore the importance of programming SSG based on sex and format preferences for optimizing exercise outcomes in sedentary.

## Introduction

Small-sided games (SSG) have become widely utilized in soccer training as a comprehensive method that combines the sport's tactics, technical skills, and physiological aspects.^1^ SSG can be adapted to specific training intensities by utilizing reduced pitch sizes, thereby accommodating variations in both acute and chronic physiological adaptations among players.^2,3^

The main aim of SSG is to simplify the complexities of a soccer match while upholding its fundamental tactical characteristics.^4^ Compared to an analytical-based approach, SSG provides a more engaging alternative that retains the dynamic essence of the sport while varying the physical and physiological stimuli.^5^ This is achieved by manipulating task constraints, including player count, game format, field dimensions, scoring regulations, and specific game rules.^1,5^ Furthermore, recreational soccer offers enhanced accessibility to the general populace, demanding fewer participants while concurrently fostering increased individual engagement.^6^ This makes recreational soccer particularly appealing, as it offers a high-intensity form of exercise that ensures participants are motivated and enjoy the game through increased individual involvement.^7^

SSG has exhibited efficacy in reducing body fat, lowering blood pressure, and enhancing cardiorespiratory, metabolic, and musculoskeletal fitness when engaged regularly.^7–9^ Similarly, SSG serves as an effective form of high-intensity interval training as it involves repeated bouts of high-intensity exercise interspersed with periods of lower intensity activity or rest.^10^ The inclusion of SSG in the routines of sedentary individuals embodies an ecological training approach that facilitates their consistent participation.^11^

Nonetheless, it is imperative to recognize that SSG exhibits dynamism and can be subject to the influence of contextual factors and tactical behaviors that manifest during gameplay.^12^ This inherent variability serves to distinguish SSG from more standardized running-based exercises.^12,13^ While the benefits of devising SSG are evident and can serve as compelling motivators for players, it is important to acknowledge that there may exist inherent variability in the stimulation and outcomes attained through these specific SSG interventions.^14^ Variability, in this context, pertains to the degree to which a set of technical actions or physiological metrics deviate or exhibit dispersion.^4^ Comprehending and effectively managing variability is of paramount significance for both coaches and researchers who aspire to maximize the efficacy of SSG in attaining their targeted training outcomes.

For instance, when closely examining the physiological and locomotor responses to SSG, it becomes evident that physiological responses demonstrate a higher degree of consistency in comparison to locomotor responses.^15^ Indeed, an observed within-player variability of approximately 2% coefficient of variation for heart rate (HR) responses is noted. In contrast, when evaluating locomotor demands, such as instances of high-speed running or sprinting, a significant level of variability emerges, with coefficients of variation exceeding 30%.^16^ SSG played in smaller formats (1v1 to 4v4), typically elicits higher HR responses compared to medium formats, such as 5v5.^17–19^ Conversely, larger pitch sizes, associated with expanded formats of play, enable players to attain greater running speeds.^20^ Consequently, it is imperative to acknowledge that the design of these games can profoundly impact the stimulus imparted to the participants.^1^

While recreational soccer for sedentary populations has garnered increasing interest, there remains a notable research gap concerning the acute responses of participants, especially regarding the variability in physiological and locomotor responses, as well as differences between various formats of play.^6,21^ Sedentary individuals exhibit distinct characteristics compared to trained players, underscoring the need for research to assess SSG programming effectiveness.^22,23^ We hypothesize that the 3v3 SSG format will consistently induce higher levels of rate of perceived exertion (RPE) compared to the 5v5 SSG format in both male and female sedentary participants. Also, it is hypothesized that locomotor demands of both SSG formats will show higher variability than physiological responses.

A comprehensive understanding of acute responses and variability within this context is crucial for researchers, coaches, and practitioners to develop more precise and effective interventions tailored to sedentary individuals participating in recreational soccer programs. Hence, this study pursues a dual objective: (a) to analyze the variability of 3v3 and 5v5 SSG formats in sedentary young adults, and (b) to compare the physiological and locomotor demands of 3v3 and 5v5 SSG formats while considering variations based on sex.

## Materials and methods

### Study design

**Figure 1.**
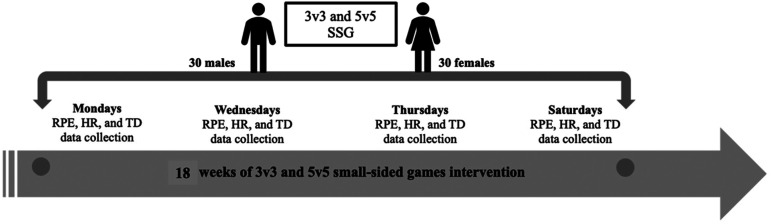
Training load monitoring during SSG intervention. Note: RPE: rate of perceived exertion; HR: heart rate; TD: total distance; SSG: small-sided games.

This study followed a longitudinal randomized controlled design, in which players were subject to monitoring regarding their training load responses during the intervention. The study commenced on 12 January 2023 and concluded on 25 May 2023. The participants actively engaged in both the 3v3 and 5v5 formats of SSG, with these sessions conducted on 5 consecutive days per week (specifically, Mondays, Wednesdays, Thursdays, Fridays, and Saturdays) spanning 18 weeks. The study's design was structured to facilitate interaction between the two SSG formats, thus furnishing a comprehensive understanding of their impact on the participants. It is pertinent to note that this study was executed in Nanchang, China. Figure 1 provides an illustrative depiction of the data collection procedures employed in this investigation.

### Participants

The sample size for this study was determined using G*Power 3.1 software (Düsseldorf, Germany). Based on a power of 0.80 and a medium effect size (ES) of 0.5, the recommended total sample size was 21 participants. To accommodate the possibility of participant attrition and to augment the study's sensitivity in detecting subtle variations, an initial recruitment effort encompassed a total of 32 subjects. Ultimately, 30 individuals (comprising 15 males and 15 females) satisfied the predetermined inclusion criteria and completed the entirety of the intervention process.

The inclusion criteria for our study were as follows: participants needed to be individuals classified as sedentary, with minimal physical activity engagement and no underlying health conditions. Additionally, participants were required to maintain a training program adherence of over 85%. Exclusion criteria stipulated that participants should not be involved in any additional professional sports training or weight loss strategies involving drugs. The mean age, height, and body mass of the participants were 20.2 ± 1.0 years, 1.67 ± 0.06 m, and 86.3 ± 11.8 kg, respectively.

All participants voluntarily enrolled and participated in both 3v3 and 5v5 SSG formats over 4 months, totaling 40 sessions per participant. Before starting, participants were provided with detailed information about potential risks or discomfort associated with the study. After being fully informed and expressing their willingness to participate, participants signed a consent form that clearly outlined the option to withdraw voluntarily from the study without penalty. The study procedures adhered to the guidelines outlined in the Declaration of Helsinki for research involving human subjects. Additionally, the study received ethical approval from the Institutional Ethical Review Board of Chengdu Institute of Physical Education, with reference code 2023#104, ensuring compliance with ethical standards for conducting and reporting the research.

### Small-sided games

Both 3v3 and 5v5 SSG formats were conducted on synthetic turf fields. The environmental conditions fluctuated during the analysis period, ranging from sessions with a temperature as low as 14°C to sessions with temperatures as high as 22°C. Additionally, the weather conditions exhibited variations, encompassing sunny periods as well as instances of light rain. Training sessions were fixed at a 1-hour duration. A standardized warm-up procedure preceded each session, encompassing 5 min of jogging, 5 min of dynamic stretching and mobility exercises, and 5 min of sprints, accelerations, and decelerations. Participants engaged in a total of 5 weekly training sessions. Sessions 1 and 5 were designated for the 3v3 format, while sessions 6 through 10 featured the 5v5 format, maintaining this sequence over the 4-month training period. Field dimensions were as follows: 20 × 30 m for 3v3, providing approximately 60 m^2^ of playing area per player, and 30 × 50 m for 5v5, offering around 150 m^2^ per player. In both formats, small goals measuring 2 × 2 m were positioned at each end.

The 3v3 training regimen included four sets lasting 2 min each, with a 2-min rest between sets. In the 5v5 format, participants engaged in 3 sets, each lasting 2 min, with a 4-min rest between sets. Table 1 summarizes the characteristics of both formats. Before formal training sessions, participants received instructions to familiarize themselves with the SSG mode, ensuring they comprehended the dynamic nature of the games and had the necessary knowledge to participate effectively. No verbal encouragement or strategic instructions were provided to players during the games. The teams were initially composed based on the monitor's assessment of technical proficiency. These team assignments remained consistent throughout the intervention period. Gender-specific teams were established, ensuring that women competed against women and men against men.

**Table 1.** Characteristics of the small-sided games training intervention.

	3v3 SSG	5v5 SSG
Goal scoring	A small goal (2 × 2 m) without goalkeeper	A small goal (2 × 2 m) without goalkeeper
Pitch size	20 × 30 m	30 × 50 m
Area per player	60 m^2^	150 m^2^
Repetitions per session	3	2
Time per repetition	4 minutes	3 minutes
Rest between repetitions	2 minutes	4 minutes
Mode of rest	stretch	Stretch
Specific rules	No offside rule	No offside rule

SSG; small-sided games.

During the games, a designated research team member was stationed at each field, responsible for promptly replacing balls when they went out of bounds. Notably, there was no verbal feedback or instructions given by the coaches during these games. Data collection was conducted by a team of researchers, all of whom held at least a master's degree in sports sciences. They were well-acquainted with the data collection procedures, having undergone prior familiarization through a pilot study before the commencement of the experimental phase. The selection of incorporating both 3v3 and 5v5 SSG stems from the consideration that the former is recognized for imposing higher mechanical work demands, whereas the latter is acknowledged for inducing a greater cumulative distance covered at different speed thresholds.

### Internal load measures

HR and RPE served as internal load indicators in all training sessions. To gauge participants’ HR intensity accurately, an HR monitor (Polar RS400, Kempele, Finland) was worn throughout each session. HRmax was determined for each participant via the Ruffier test.^24^ HR readings were taken intermittently during sessions and at the session's end, with the highest recorded as the data point. Perceived exertion levels were evaluated using the Borg scale CR10,^25^ which spans from 0 (Nothing at all) to 10 (Very, very hard - maximum exertion). Participants promptly rated their perceived exertion using the Borg scale CR10 after each session.

An expert researcher conducted HRmax recording and calculation, ensuring data precision. The researcher's reliability was assessed to maintain data consistency. Subsequently, Borg scale CR10 scores and statistical analysis were carried out by a university physical education instructor following each training session.

### External load measures

To quantify the external load, we employed a portable 10 Hz VX Motion SGPS unit (VX Motion, Wellington, New Zealand) to capture the total distance (TD) traversed by each participant in every session. The measurement methodology employed by this instrument has been substantiated for its effectiveness and reliability in prior research endeavors.^26^ The recorded TD encompassed the distance covered during walking, interval running, and sprinting activities on the field. To ensure data accuracy for comparative analysis across distinct SSG formats, intermittent rest intervals were purposefully omitted from the TD calculations.

### Statistical procedures

Descriptive statistics, including mean and standard deviation, were used to present the data. Within-player variability and between-player variability were expressed as the coefficient of variation (%). The variation of outcomes across the sessions was tested using repeated measures ANOVA, with ES identified using partial eta squared. Pairwise comparisons were conducted using Bonferroni's test, and the ES was calculated using Cohen's d. To compare the formats of play, a univariate ANOVA was performed, considering the interaction between sex and the format of play. The ES for pairwise comparisons was calculated using Cohen's d. All statistical analyses were conducted using SPSS software (version 28.0.0.0, IBM, New York, USA), with a significance level set at p < 0.05.

## Results

Table 2 displays the coefficient of variability of TD, HR, and RPE responses for both male and female participants across different formats of play, specifically 3v3 and 5v5. The within-player variability in TD for the 3v3 format ranged from 10.1% to 5.6%, while for the 5v5 format, it ranged from 6.4% to 5.5% across the study months. Female participants showed similar variability, with TD ranging from 10.3% to 5.2% for the 3v3 format and 6.2% to 5.0% for the 5v5 format. The within-player HR for the 3v3 format varied between 2.5% and 1.8%, and for the 5v5 format, it ranged from 2.8% to 1.6% across the study months. Female participants exhibited comparable variability, with HR ranging from 2.6% to 1.8% for the 3v3 format and 2.7% to 1.4% for the 5v5 format. Regarding the RPE, within-player variability for the 3v3 format spanned from 10.8% to 5.0%, and for the 5v5 format, it ranged from 17.1% to 5.4% across the study months. Female participants displayed a similar pattern, with RPE variability ranging from 11.5% to 5.7% for the 3v3 format and 17.6% to 4.9% for the 5v5 format.

**Table 2.** Coefficient of variation comparison by formats of play (3v3 and 5v5) and sex (male and female).

		TD within-player CV%	TD between-player CV%	TD CV%	HR within-player CV%	HR between- player CV%	HR CV%	RPE within-player CV%	RPE between-player CV%	RPE CV%
	SSG format	Month 1								
Males	3v3	10.1	10.4	10.3 ± 0.2	2.2	2.2	2.2 ± 0	5.0	4.9	5.0 ± 0.1
	5v5	6.8	8.0	7.4 ± 0.8	2.3	2.2	2.3 ± 0.1	5.4	4.9	5.2 ± 0.4
Females	3v3	10.3	11.0	10.7 ± 0.5	2.6	2.6	2.6 ± 0	5.7	6.0	5.9 ± 0.1
	5v5	6.2	6.7	6.5 ± 0.4	2.3	2.2	2.3 ± 0.1	4.9	5.0	5.0 ± 0.1
		Month 2								
Males	3v3	5.6	6.1	5.9 ± 0.4	2.5	2.1	2.3 ± 0.3	10.8	7.3	9.1 ± 2.5
	5v5	5.5	5.7	5.6 ± 0.1	2.8	1.8	2.3 ± 0.7	17.1	7.1	12.1 ± 7.1
Females	3v3	5.2	5.4	5.3 ± 0.1	2.5	2.2	2.4 ± 0.2	11.5	8.0	9.8 ± 2.5
	5v5	5.1	5.1	5.1 ± 0	2.7	1.8	2.3 ± 0.6	17.6	7.8	12.7 ± 7.0
		Month 3								
Males	3v3	6.4	6.6	6.5 ± 0.1	1.8	1.8	1.8 ± 0	7.2	5.6	6.4 ± 1.1
	5v5	6.4	7.0	6.7 ± 0.4	1.6	1.7	1.7 ± 0.1	5.4	5.3	5.4 ± 0.1
Females	3v3	5.7	5.3	5.5 ± 0.3	1.8	1.7	1.8 ± 0.1	8.1	6.2	7.2 ± 1.3
	5v5	5.0	5.0	5.0 ± 0	1.4	1.4	1.4 ± 0	6.8	6.0	6.4 ± 0.6
		Month 4								
Males	3v3	6.1	6.3	6.2 ± 0.1	1.9	1.9	1.9 ± 0	8.9	6.9	7.9 ± 1.4
	5v5	5.6	5.9	5.8 ± 0.2	2.0	1.6	1.8 ± 0.3	14.7	8.6	11.7 ± 4.3
Females	3v3	5.2	5.1	5.2 ± 0.1	1.8	1.8	1.8 ± 0	9.1	7.0	8.1 ± 1.5
	5v5	5.5	5.5	5.5 ± 0	2.0	1.8	1.9 ± 0.1	14.2	8.1	11.2 ± 4.3

TD: total distance; HR: heart rate RPE: rate of perceived exertion; CV%: percentage of the coefficient of variation; SSG: small-sided games.

Considering between-players variability, the TD for the 3v3 format showed variations between 10.4% and 6.1%, while for the 5v5 format, it ranged from 8.0% to 5.7% across the study months. Female participants exhibited comparable variability, with TD ranging from 11.0% to 5.1% for the 3v3 format and 6.7% to 5.0% for the 5v5 format. The between-players HR variability for the 3v3 format varied between 2.2% and 1.8%, and for the 5v5 format, it ranged from 2.2% to 1.6% across the study months. Female participants displayed variability ranging from 2.6% to 1.7% for the 3v3 format and 2.2% to 1.4% for the 5v5 format. Regarding RPE, between-players variability for the 3v3 format spanned from 7.3% to 4.9%, and for the 5v5 format, it ranged from 8.6% to 4.9% across the study months. Female participants showed a similar pattern, with RPE variability ranging from 8.0% to 6.0% for the 3v3 format and 8.1% to 5.0% for the 5v5 format.

The analysis of HR and RPE involved repeated measures of ANOVA for 10 sessions per format (3v3 and 5v5) conducted in each training month, categorized by sex (females and males) (Table 3).

**Table 3.** Descriptive statistics (mean ± standard deviation) of total distance, HR, and RPE of each format of play (3v3 and 5v5) within each monthly analysis for males and females.

	SSG format	TD (mean ± SD)	HR (mean ± SD)	RPE (mean ± SD)	TD (mean ± SD)	HR (mean ± SD)	RPE (mean ± SD)	TD (mean ± SD)	HR (mean ± SD)	RPE (mean ± SD)	TD (mean ± SD)	HR (mean ± SD)	RPE (mean ± SD)
		Month 1	Month 1	Month 1	Month 2	Month 2	Month 2	Month 3	Month 3	Month 3	Month 4	Month 4	Month 4
M	3v3^a^	2.71 ± 0.02	190.0 ± 0.4	8.3 ± 0.1	2.79 ± 0.02	183.8 ± 0.3	6.8 ± 0.1	3.04 ± 0.03	186.1 ± 0.1	7.8 ± 0.1	3.19 ± 0.02	183.5 ± 0.3	6.9 ± 0.1
	5v5^a^	2.79 ± 0.02	190.0 ± 0.2	7.9 ± 0.1	2.82 ± 0.02	181.8 ± 0.3	6.3 ± 0.1	3.04 ± 0.02	187.4 ± 0.3	8.1 ± 0.1	3.36 ± 0.06	184.7 ± 0.3	6.8 ± 0.1
F	3v3^a^	2.43 ± 0.01	188.3 ± 0.4	8.4 ± 0.1	2.59 ± 0.02	183.4 ± 0.4	6.9 ± 0.1	2.85 ± 0.01	186.3 ± 0.3	7.9 ± 0.1	3.00 ± 0.01	183.4 ± 0.2	7.0 ± 0.1
	5v5^a^	2.59 ± 0.01	187.9 ± 0.3	7.9 ± 0.1	2.61 ± 0.01	181.9 ± 0.2	6.3 ± 0.1	2.83 ± 0.01	186.9 ± 0.3	8.1 ± 0.1	3.09 ± 0.01	184.0 ± 0.3	6.8 ± 0.1

M: males; F: females; TD: total distance; HR: heart rate; RPE: rate of perceived exertion; SSG: small-sided games; ^a^Repeated measures ANOVA conducted for all the training sessions analyzed within which month.

During the first month, significant HR differences were observed for females in the 5v5 format (F = 2.231, *p* = 0.024, ηp2 = 0.137). In the second month, males exhibited significant HR variation in both 3v3 (F = 7.571, *p* < 0.001, ηp2 = 0.351) and 5v5 (F = 22.752, *p* < 0.001, ηp2 = 0.619) formats. Similarly, for females, significant HR differences were observed in both 3v3 (F = 6.236, *p* < 0.001, ηp2 = 0.308) and 5v5 (F = 18.210, *p* < 0.001, ηp2 = 0.565) formats. Both males and females exhibited significant RPE variation in both 3v3 (Males: F = 20.910, *p* < 0.001, ηp2 = 0.599; Females: F = 20.510, *p* < 0.001, ηp2 = 0.594) and 5v5 (Males: F = 76.362, *p* < 0.001, ηp2 = 0.845; Females: F = 69.188, *p* < 0.001, ηp2 = 0.832) formats.

Moving to the third month, males demonstrated significant RPE variation in both 3v3 (F = 12.004, *p* < 0.001, ηp2 = 0.462) and 5v5 (F = 5.702, *p* < 0.001, ηp2 = 0.289) formats. For females, significant RPE differences were also observed in both 3v3 (F = 12.343, *p* < 0.001, ηp2 = 0.469) and 5v5 (F = 6.776, *p* < 0.001, ηp2 = 0.326) formats. Additionally, males demonstrated significant TD variation in 3v3 format (F = 3.496, *p* = 0.001, ηp2 = 0.200). For females, significant TD differences were observed in both 3v3 (F = 4.475, *p* < 0.001, ηp2 = 0.242) and 5v5 (F = 2.263, *p* *=* 0.022, ηp2 = 0.139) formats.

In the fourth month, significant HR variation was observed for females in the 5v5 format (F = 5.188, *p* < 0.001, ηp2 = 0.270). Males exhibited significant RPE variation in both 3v3 (F = 11.544, *p* < 0.001, ηp2 = 0.452) and 5v5 (F = 34.916, *p* < 0.001, ηp2 = 0.714) formats. Similarly, for females, significant RPE differences were found in both 3v3 (F = 12.319, *p* < 0.001, ηp2 = 0.468) and 5v5 (F = 33.785, *p* < 0.001, ηp2 = 0.707) formats.

Table 4 displays mean values (± standard deviation) for sessions in both 3v3 and 5v5 formats, stratified by sex. To investigate the interaction between sex and format concerning the HR variable, we conducted univariate analysis. The results indicated a significant interaction (F = 3.537, *p* = 0.020, ηp2 = 0.159).

**Table 4.** Descriptive statistics (mean ± standard deviation) for the entire sessions performed in each format of play (3v3 and 5v5), while considering the sexes.

	3v3 Mean ± SD	5v5 Mean ± SD	*p*-value (between formats)	*p*-value (between sexes)
Males				
Total distance (m)	2.93 ± 0.05	3.00 ± 0.05	*p* < 0.001 | ES = 1.4	3v3: *p* < 0.001 | ES = 5.1
				5v5: *p* < 0.001 | ES = 5.3
HR (bpm)	185.7 ± 0.6	185.6 ± 0.4	*p* = 0.617 | ES = 0.2	3v3: *p* = 0.053 | ES = 0.7
				5v5: *p* = 0.020 | ES = 0.9
RPE (A.U.)	7.5 ± 0.1	7.3 ± 0.1	*p* < 0.001 | ES = 2.0	3v3: *p* = 0.006 | ES = 1.0
				5v5: *p* = 0.628 | ES = 0
Females				
Total distance (m)	2.72 ± 0.03	2.78 ± 0.03	*p* < 0.001 | ES = 2.0	3v3: *p* < 0.001 | ES = 5.1
				5v5: *p* < 0.001 | ES = 5.3
HR (bpm)	185.3 ± 0.6	185.2 ± 0.5	*p* = 0.363 | ES = 0.2	3v3: *p* = 0.053 | ES = 0.7
				5v5: *p* = 0.020 | ES = 0.9
RPE (A.U.)	7.6 ± 0.1	7.3 ± 0.1	*p* < 0.001 | ES = 3.0	3v3: *p* = 0.006 | ES = 1.0
				5v5: *p* = 0.628 | ES = 0

HR: heart rate; RPE: rate of perceived exertion; ES: effect size.

Among male participants, HR did not significantly differ between 3v3 and 5v5 formats (185.7 ± 0.6 vs. 185.6 ± 0.4 bpm, respectively; *p* = 0.617, ES = 0.2). Similarly, among female participants, there was no significant difference in HR between the 3v3 and 5v5 formats (185.3 ± 0.6 vs. 185.2 ± 0.5 bpm, respectively; *p* = 0.362, ES = 0.2). Additionally, within the 3v3 format, there were no significant differences in HR between sexes (*p* = 0.053, ES = 0.7). However, within the 5v5 format, significant sex-based differences in HR were observed (*p* = 0.020, ES = 0.9). For the RPE variable, we conducted univariate analysis to explore the interaction between sex and format. The results demonstrated a significant interaction (F = 41.728, *p* < 0.001, ηp2 = 0.691).

Among male participants, RPE significantly differed between 3v3 and 5v5 formats (7.5 ± 0.1 vs. 7.3 ± 0.1 A.U., respectively; *p* < 0.001, ES = 2.0). Similarly, among female participants, RPE significantly varied between 3v3 and 5v5 formats (7.6 ± 0.1 vs. 7.3 ± 0.1 A.U., respectively; *p* < 0.001, ES = 3.0). Furthermore, when comparing sexes within the 3v3 format, men reported significantly higher RPE levels than women (*p* = 0.006, ES = 1.0). However, within the 5v5 format, there were no significant sex-based differences in RPE (*p* = 0.628, ES = 0). For the TD variable, univariate analysis examined the interaction between sex and format. The results showed a significant interaction (F = 150.032, *p* < 0.001, ηp2 = 0.889).

Among male participants, TD significantly differed between 3v3 and 5v5 formats (2.93 ± 0.05 vs. 3.00 ± 0.05 m, respectively; *p* < 0.001, ES = 1.4). Similarly, among female participants, TD significantly varied between 3v3 and 5v5 formats (2.72 ± 0.03 vs. 2.78 ± 0.03 m, respectively; *p* < 0.001, ES = 2.0). Furthermore, when comparing sexes within the 3v3 format, men covered significantly greater distances than women (*p* < 0.001, ES = 5.1). Likewise, within the 5v5 format, significant sex-based differences in TD were observed (*p* < 0.001, ES = 5.3).

## Discussion

This study aimed to investigate the variability of different forms of recreational SSG in a sedentary population and compare the physiological and exercise demands of these different SSG formats. The results showed less variability between players and within players in HR, indicating consistent responses to the SSG formats. However, higher variability was observed in the RPE in both SSG formats, indicating individual differences in perceived effort. Furthermore, comparisons were made between the performances in different SSG formats. The results revealed that the 5v5 format resulted in higher HR than the 3v3 format for both male and female participants.

Variability analysis of different SSG formats is a commonly used test among sports scientists and coaches to assess the reproducibility of the training stimulus in participants.^4^ Given that SSG are frequently used by coaches to provide a physical stimulus, it is crucial to understand the between- and within-player variability that can occur in different SSG formats to ensure consistent implementation of an appropriate stimulus over time.^27^ Previous studies have demonstrated that TD covered during SSG exhibits small-to-moderate within-session variability, with coefficients of variation (%CV) ranging from approximately 5% to 9%, and 1% to 10% change between the lowest and highest sets/repetitions across various SSG formats such as 1v1, 2v2, 3v3, 4v4, 5v5, and 7v7.^24,25^ Consistent with these findings, our study observed similar results in terms of within-player variability. The TD covered exhibited variations within the range of 10.1% to 5.6% for the 3v3 format and 6.4% to 5.5% for the 5v5 format across the analyzed months. For female participants, the variability in TD ranged from 10.3% to 5.2% for the 3v3 format and 6.2% to 5.0% for the 5v5 format, respectively.

The observed variability in TD measures in the study could be attributed to several factors. Firstly, individual participant characteristics, such as baseline fitness levels, movement abilities, and overall physical condition, can significantly influence their performance and exertion during the SSG.^28^ Participants with higher baseline fitness may cover more distance, while those with limited physical capabilities may exhibit variations in TD.^29^ Additionally, the design of the SSG, particularly the formats with different team sizes or rule modifications, may lead to differences in participants’ involvement and spatial awareness, impacting their movement patterns and resulting in fluctuations in TD.^30^

When considering the RPE,^31^ higher variabilities were observed. Consistent with findings from a systematic review, large within-session variability was found for RPE. Specifically, in the 5v5 format, both male and female participants exhibited RPE variations within the range of 17.1% to 5.4%.^32^ In contrast, there was less variability between players of both genders, with the RPE ranging from 7.3% to 4.9% for the 3v3 format. Interestingly, within-player variability was greater than between-player variability for RPE in SSG formats.^33^ Considering potential gender-related physiological differences may further enrich the interpretation of the results, leading to more targeted and personalized training approaches for different populations.

The larger RPE within-session variability may be influenced by individual differences in perception and subjective feelings of effort during the SSGs. Sedentary individuals can show varying levels of experience and familiarity with physical activity, leading to differences in how they perceive and rate their exertion levels.^34^ Additionally, fitness levels, could once again, contribute to the observed variability in RPE. Participants with lower fitness levels or those new to physical activity might find the games more challenging, resulting in higher perceived exertion.^35^ Moreover, the specific rules, intensity, and duration of each SSG format may elicit different responses in participants, further contributing to the variability in RPE.

As for HR responses, the more stable pattern observed could be due to the controlled and structured nature of the SSGs. Since the study was conducted on sedentary individuals, their physiological responses might be less variable compared to trained athletes.^34^ The relatively consistent HR patterns may be a reflection of the participants’ limited cardiovascular adaptations to exercise at the onset of the study.^27^ As participants engaged in similar SSG formats, the cardiovascular demands might not have varied significantly, resulting in relatively stable HR responses within and between players. Additionally, the training status and baseline fitness of the sedentary population might play a role in the HR responses, with less variation observed compared to previous studies conducted on trained athletes.^36^

In the present study, the 3v3 format showed a variation of 2.5% to 1.8% in the coefficient of variation within players of both genders, while the 5v5 format ranged from 2.7% to 1.4%. Similarly, between-player variability exhibited similar average values for both males and females. These values were slightly lower than those reported in a previous study investigating the variability of HR responses in the same SSG formats.^34^ For instance, in the 3v3 format, previous studies reported a variation ranging from 6.0% to 6.5% in the coefficient of variation, while for the 5v5 format, the range was 10.3% to 10.4%.^34,36^ Coaches and practitioners should be aware of these variabilities to effectively manage and monitor the physiological and perceived exertion demands placed on sedentary participants during SSG training sessions.

The second aim of this study was to compare different SSG formats. While there are some physiological differences between males and females, especially in terms of muscle mass and hormonal profiles, the overall cardiovascular responses to exercise can still be relatively similar.^37^ However, significant differences in both TD and RPE were observed between the 3v3 and 5v5 formats for both males and females. Similar results were observed when comparing the sexes.^38^ In that study, male soccer players covered a significantly greater TD that was 24.7% greater compared to females.^38^ However, this study involved professional soccer players, using the 4v4 format. Given the lack of studies conducted on sedentary individuals, direct comparisons are challenging due to methodological differences.

This study has several limitations that should be acknowledged. It's important to acknowledge that our study's sample consisted of volunteers recruited from universities. This recruitment approach may introduce biases related to the age, fitness levels, and motivation of participants. These biases could potentially affect the generalizability of our findings. For instance, the relatively young age and potential higher motivation to participate among university students might have influenced their responses to SSG differently than a more diverse sedentary population. We recognize that these biases might have led to specific directions of influence on our results, such as potentially underestimating or overestimating the observed physiological responses.

Nevertheless, this study offers practical applications for sedentary recreational populations participating in recreational soccer SSG. Firstly, incorporating both male and female participants in our study reflects the real-world inclusivity of recreational soccer programs, ensuring our findings apply broadly. It also allows us to investigate potential sex-specific differences in responses to SSGs, which can be vital for tailored training programs. Our goal is to offer practical insights for optimizing recreational soccer for sedentary individuals, making it safe and effective for everyone, regardless of sex. Secondly, by recognizing the reproducibility of physiological responses in both 3v3 and 5v5 SSG formats, trainers can design exercise routines that cater to the preferences and capabilities of sedentary individuals. The study indicates that the 3v3 format consistently induced higher levels of perceived exertion. Therefore, coaches may opt for this format when aiming to challenge participants’ perceived effort and stimulate greater physiological responses. The use of both 3v3 and 5v5 SSG formats can be integrated into training programs as effective methods for improving fitness levels and promoting physical activity engagement in sedentary populations.

## Conclusions

This study aimed to analyze the differences between 3v3 and 5v5 formats in sedentary recreational populations engaged in soccer SSGs. The results suggest that both formats are feasible for this population, with no significant variability within or between players. These findings underscore the significance of coaches acknowledging variability in sedentary recreational populations and comprehending distinctions among SSG formats. This awareness is vital for successfully implementing various SSG formats that align with participants’ physiological needs. Additionally, coaches should contemplate integrating suitable techniques and strategies to augment participant engagement and enjoyment during the games.
